# TGF-Beta Induces Serous Borderline Ovarian Tumor Cell Invasion by Activating EMT but Triggers Apoptosis in Low-Grade Serous Ovarian Carcinoma Cells

**DOI:** 10.1371/journal.pone.0042436

**Published:** 2012-08-15

**Authors:** Jung-Chien Cheng, Nelly Auersperg, Peter C. K. Leung

**Affiliations:** Department of Obstetrics and Gynecology, Child and Family Research Institute, University of British Columbia, Vancouver, British Columbia, Canada; The University of Kansas Medical Center, United States of America

## Abstract

Apoptosis in ovarian surface epithelial (OSE) cells is induced by transforming growth factor-beta (TGF-β). However, high-grade serous ovarian carcinomas (HGC) are refractory to the inhibitory functions of TGF-β; their invasiveness is up-regulated by TGF-β through epithelial-mesenchymal transition (EMT) activation. Serous borderline ovarian tumors (SBOT) have been recognized as distinct entities that give rise to invasive low-grade serous carcinomas (LGC), which have a relatively poor prognosis and are unrelated to HGC. While it is not fully understood how TGF-β plays disparate roles in OSE cells and its malignant derivative HGC, its role in SBOT and LGC remains unknown. Here we demonstrate the effects of TGF-β on cultured SBOT3.1 and LGC-derived MPSC1 cells, which express TGF-β type I and type II receptors. TGF-β treatment induced the invasiveness of SBOT3.1 cells but reduced the invasiveness of MPSC1 cells. The analysis of apoptosis, which was assessed by cleaved caspase-3 and trypan blue exclusion assay, revealed TGF-β-induced apoptosis in MPSC1, but not SBOT3.1 cells. The pro-apoptotic effect of TGF-β on LGC cells was confirmed in another immortalized LGC cell line ILGC. TGF-β treatment led to the activation of Smad3 but not Smad2. The specific TβRI inhibitor SB431542 and TβRI siRNA abolished the SBOT3.1 invasion induced by TGF-β, and it prevented TGF-β-induced apoptosis in MPSC1 cells. In SBOT3.1 cells, TGF-β down-regulated E-cadherin and concurrently up-regulated N-cadherin. TGF-β up-regulated the expression of the transcriptional repressors of E-cadherin, Snail, Slug, Twist and ZEB1. In contrast, co-treatment with SB431542 and TβRI depletion by siRNA abolished the effects of TGF-β on the relative cadherin expression levels and that of Snail, Slug, Twist and ZEB1 as well. This study demonstrates dual TGF-β functions: the induction of SBOT cell invasion by EMT activation and apoptosis promotion in LGC cells.

## Introduction

Transforming growth factor-beta (TGF-β) is a pleiotropic cytokine that regulates cell proliferation, apoptosis, differentiation, migration and invasion [1]. TGF-β signals through transmembrane type I (TβRI) and type II (TβRII) receptors to initiate downstream signaling [2]. In the canonical pathway, TGF-β binding to TβRII recruits and phosphorylates TβRI, which results in TβRI activation. Activated TβRI phosphorylates the receptor-regulated Smad proteins Smad2 and Smad3. Phosphorylated Smad2 and Smad3 then co-associate with Smad4, translocate into the nucleus and regulate gene expression by binding to Smad-specific binding elements in the promoters of TGF-β-regulated genes [3]. In humans, TGF-β overexpression has been detected in many cancer types and correlates with tumor metastasis, progression and prognosis [4], [5]. Many studies have indicated that TGF-β can function as a tumor suppressor and promoter depending on the context [6]. TGF-β acts as a tumor suppressor by inhibiting cell proliferation, while as a tumor promoter, TGF-β induces an epithelial-mesenchymal transition (EMT), cell motility and invasion [7].

EMT has been recognized as a key process for embryonic development and metastasis [8]. Cells undergoing EMT down-regulate the expression of the E-cadherin epithelial marker and increase the expression of N-cadherin, a mesenchymal marker. This process has been shown proceed through a set of transcription factors including the Snail and Slug zinc-finger proteins, the Twist bHLH factor and the ZEB1 zinc-finger protein [9]. TGF-β is a potent inducer of EMT, which was first recognized in cultured normal mammary epithelial cells [10]. TGF-β can induce EMT by activating Smad-dependent and Smad-independent pathways [11]. Ectopic expression of Smad2 or Smad3 with Smad4 enhances EMT, whereas ectopic expression of dominant-negative Smad2, Smad3 or Smad4 blocks TGF-β-induced EMT [12].

TGF-β acts as a tumor suppressor in the early stages of cancer progression, and it becomes a tumor promoter in later stages [5]. TGF-β1, TGF-β2 and TGF-β3 overexpression has been reported in human ovarian tumors [13]. Ovarian cancer is thought to arise from normal ovarian surface epithelium (OSE) [14]. TGF-β has been shown to inhibit human OSE proliferation and induce apoptosis, which may prevent the over-proliferation of cells during a normal ovulatory cycle [15]. In the later stages of ovarian cancer, TGF-β enhances tumor cell proliferation and promotes metastasis by inducing an EMT [16], [17].

It has recently been recognized that high-grade serous ovarian carcinoma (HGC) and low-grade serous ovarian carcinoma (LGC) are fundamentally different types of tumors that develop from distinct molecular pathways [18]. Compared with HGC, LGC accounts for a small proportion (9%) of all serous ovarian carcinomas [19]. Invasive LGC is developed from non-invasive borderline serous ovarian tumors (SBOT) [20], [21]. In ovarian cancer, TGF-β-induced EMT is believed to play an important role in the regulation of cell invasion and metastasis [22]. It has been shown that TGF-β and TβRII are expressed in primary human borderline ovarian tumors [13]. Although the function of TGF-β in HGC has been extensively investigated, to our knowledge, no study has examined the effects of TGF-β in the SBOT/LGC system. Our recent studies demonstrate that E-cadherin down-regulation induces SBOT cell invasion, suggesting that EMT is involved in the progression from non-invasive SBOT to invasive LGC [23]–[25]. Thus, this study was undertaken to test the hypothesis that TGF-β induces SBOT invasion by activating EMT.

## Materials and Methods

### Cell culture

The SBOT3.1 [26] and SV40 LT/ST immortalized LGC (ILGC) [27] cell lines were established in our laboratory. SBOT and ILGC cells were grown in a 1∶1 (v/v) mixture of M199/MCDB105 medium (Sigma, Oakville, ON) supplemented with 10% fetal bovine serum (FBS; Hyclone Laboratories Inc., Logan, UT). The MPSC1 cell line, which was established from a LGC (provided by Dr. Ie-Ming Shih, Department of Pathology, Johns Hopkins Medical Institutions, Baltimore, MD), was maintained in RPMI 1640 (Invitrogen, Burlington, ON) supplemented with 10% FBS [28]. Cultures were maintained at 37°C in a humidified 5% CO_2_ atmosphere in air.

### Antibodies and reagents

The polyclonal anti-β-actin antibody was obtained from Santa Cruz Biotechnology (Santa Cruz, CA). The monoclonal anti-E-cadherin and anti-N-cadherin antibodies were obtained from BD Biosciences (Mississauga, ON). The monoclonal anti-phospho-Smad3, anti-Smad3, anti-Smad2, polyclonal anti-TGF-β receptor I, anti-TGF-β receptor II, anti-phospho-Smad2, anti-Smad4 and anti-caspase-3 antibodies were obtained from Cell Signaling Technology (Danvers, MA). Horseradish peroxidase-conjugated goat anti-mouse IgG and goat anti-rabbit IgG were obtained from Bio-Rad Laboratories (Hercules, CA). Horseradish peroxidase-conjugated donkey anti-goat IgG was obtained from Santa Cruz Biotechnology. Recombinant human TGF-β1 was obtained from R&D Systems (Minneapolis, MN). SB431542 was obtained from Sigma.

### Small interfering RNA (siRNA) transfection

To knock down endogenous TGF-β receptor I (TβRI), cells were transfected with 50 nM ON-TARGET*plus* SMART*pool* TβRI siRNA (Dharmacon, Lafayette, CO) using Lipofectamine RNAiMAX (Invitrogen, Burlington, ON). The siCONTROL non-targeting siRNA pool (Dharmacon) was used as a transfection control. The knockdown efficiency was examined by RT-qPCR or western blot analysis.

### Western blot

Equal protein amounts were separated by SDS-polyacrylamide gel electrophoresis and transferred to PVDF membranes. The membranes were blocked with Tris-buffered saline containing 5% non-fat dry milk for 1 hr. The membranes were then incubated overnight at 4°C with primary antibodies followed by incubation with HRP-conjugated secondary antibodies. Immunoreactive bands were detected using an enhanced chemiluminescent substrate. The membranes were stripped with stripping buffer at 50°C for 30 min and reprobed with anti-β-actin as a loading control.

### Reverse transcription quantitative real-time PCR (RT-qPCR)

Total RNA was extracted using TRIzol reagent (Invitrogen) according to the manufacturer's instructions. Reverse transcription was performed using 3 µg RNA, random primers and M-MLV reverse transcriptase (Promega, Madison, WI). The primers used for SYBR Green reverse transcription-qPCR (RT-qPCR) were as follows: TβRI: 5′-GTT AAG GCC AAA TAT CCC AAA CA-3′ (sense) and 5′- ATA ATT TTA GCC ATT ACT CTC AAG G-3′ (antisense); E-cadherin: 5′-ACA GCC CCG CCT TAT GAT T-3′ (sense) and 5′-TCG GAA CCG CTT CCT TCA-3′ (antisense); N-cadherin: 5′-GGA CAG TTC CTG AGG GAT CA-3′ (sense) and 5′-GGA TTG CCT TCC ATG TCT GT-3′ (antisense); Snail: 5′-CCC CAA TCG GAA GCC TAA CT-3′ (sense) and 5′-GCT GGA AGG TAA ACT CTG GAT TAG A-3′ (antisense); Slug: 5′-TTC GGA CCC ACA CAT TAC CT-3′ (sense) and 5′-GCA GTG AGG GCA AGA AAA AG-3′ (antisense); Twist: 5′-GGA GTC CGC AGT CTT ACG AG-3′ (sense) and 5′-TCT GGA GGA CCT GGT AGA GG-3′ (antisense); ZEB1: 5′-GCA CCT GAA GAG GAC CAG AG-3′ (sense) and 5′-TGC ATC TGG TGT TCC ATT TT-3′ (antisense); and GAPDH: 5′-GAG TCA ACG GAT TTG GTC GT-3′ (sense) and 5′-GAC AAG CTT CCC GTT CTC AG-3′ (antisense). RT-qPCR was performed using the Applied Biosystems 7300 Real-Time PCR System (Perkin-Elmer), which was equipped with a 96-well optical reaction plate. All RT-qPCR experiments were performed in triplicate, and the mean value was used for to determine the mRNA levels. Relative quantification of the mRNA levels was performed using the comparative Ct method with GAPDH as the reference gene and using the 2^−ΔΔCt^ formula.

### Transwell invasion assay

Invasion assays were performed in Boyden chambers with minor modifications [29]. Cell culture inserts (24-well, 8 µm pore size; BD Biosciences, Mississauga, ON) were seeded with 1×10^5^ cells in 250 µl of medium supplemented with 0.1% FBS. Inserts pre-coated with growth factor-reduced Matrigel (40 µl, 1 mg/ml, BD Biosciences) were used for the invasion assays. Medium supplemented with 10% FBS (750 µl) was added to the lower chamber and served as a chemotactic agent. After 48 hr incubation, non-invading cells were removed from the upper side of the membrane. Cells that penetrated the membrane were fixed with cold methanol, and cell nuclei were stained with Hoechst 33258 and counted by epifluorescence microscopy using Northern Eclipse 6.0 software (Empix Imaging, Mississauga, ON). Triplicate inserts were used for each individual experiment.

### Statistical analysis

Results are presented as the mean ± SEM of at least three independent experiments and were analyzed by the one-way ANOVA test followed by Tukey's test using PRISM software. A *p*<0.05 was considered statistically significant.

## Results

### TGF-β induces SBOT3.1 cell invasion but decreases the invasion of MPSC1 cells

**Figure 1:**
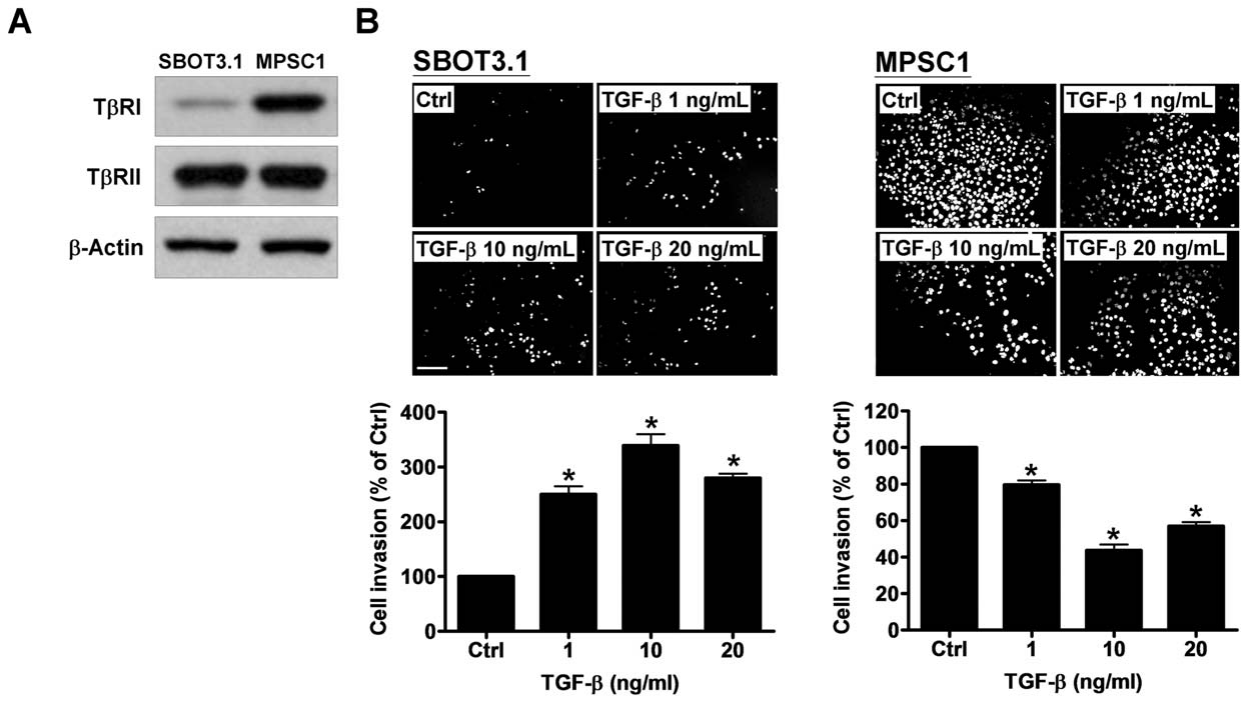
TGF-β induces SBOT3.1 cell invasion but reduces the invasiveness of MPSC1 cells. A, The endogenous protein levels of TGF-β type I receptor (TβRI) and type II receptor (TβRII) in SBOT3.1 and MPSC1 cells were analyzed by western blot. B, SBOT3.1 and MPSC1 cells were treated with increasing TGF-β doses (1, 10 and 20 ng/ml) and seeded into Matrigel-coated transwell inserts. After 48 hr incubation, non-invading cells were wiped from the upper side of the filter, and the nuclei of the invading cells were stained with Hoechst 33258. The top panels show representative images of the invasion assays. The scale bar represents 200 µm. The bottom panels show summarized quantitative results, which are expressed as the mean ± SEM of at least three independent experiments. *p<0.05 compared with the control samples (Ctrl).

To test the hypothesis that TGF-β affects SBOT and LGC cell invasion by activating EMT, we used SBOT3.1 and a LGC-derived cell line MPSC1. Western blot analysis demonstrated that TGF-β type I (TβRI) and type II (TβRII) were expressed in SBOT3.1 and MPSC1 cells (Figure 1A). The TβRI expression level was higher in MPSC1 cells than SBOT3.1 cells although no significant difference was observed for the TβRII expression levels (Figure 1A). To examine the effect of TGF-β on cell invasion, Matrigel-coated transwells were used. As shown in Figure 1B, SBOT3.1 cells were non-invasive, whereas MPSC1 cells were highly invasive. Treatment with TGF-β (1, 10 and 20 ng/ml) significantly induced SBOT cell invasion. Surprisingly, TGF-β treatment significantly decreased the invasiveness of MPSC1 cells. The maximal effect of TGF-β was observed at 10 ng/ml in both cases (Figure 1B).

### TGF-β induces apoptosis in MPSC1, but not in SBOT3.1 cells

**Figure 2:**
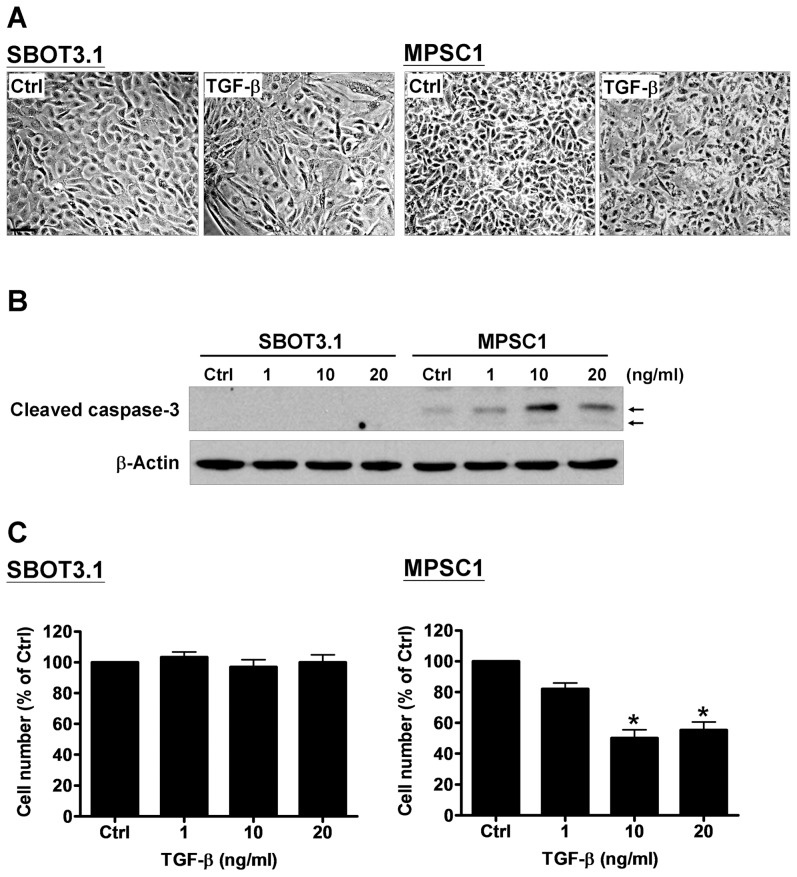
TGF-β induces apoptosis in MPSC1 but not SBOT3.1 cells. A, SBOT3.1 and MPSC1 cells were treated without (control; Ctrl) or with 10 ng/ml TGF-β for 48 hr, and the resultant morphology was microscopically examined. The scale bar represents 200 µm. B, SBOT3.1 and MPSC1 cells were treated with increasing TGF-β doses (1, 10 and 20 ng/ml) for 24 hr, and the levels of cleaved caspase-3 were examined using western blot analysis. Arrows indicate cleaved caspase-3. C, SBOT3.1 and MPSC1 cells were treated with increasing TGF-β doses (1, 10 and 20 ng/ml) for 48 hr, and the cell number changes were examined using a trypan blue exclusion assay. *p<0.05 compared with the control samples (Ctrl).

To investigate the possible mechanism that mediated the inhibitory effect of TGF-β on MPSC1 cell invasion, we examined the TGF-β effect on apoptosis in SBOT3.1 and MPSC1 cells. TGF-β treatment for 48 hr induced EMT-like morphological changes in SBOT3.1 cells from a cobblestone-like morphology to a fibroblastic-spindle shape. In contrast, the number of cells was lower in TGF-β-treated MPSC1 cells, suggesting that TGF-β had a pro-apoptotic effect (Figure 2A). To further confirm this result, the cleaved caspase-3 levels were examined after TGF-β treatment. As shown in Figure 2B, TGF-β treatment for 24 hr did not affect the expression of cleaved caspase-3 in SBOT3.1 cells. However, TGF-β induced cleaved caspase-3 expression in MPSC1 cells. Furthermore, the pro-apoptotic effect of TGF-β on SBOT3.1 and MPSC1 cells was examined using a trypan blue exclusion assay. Similar to the results obtained from the cleaved caspase-3 assay, TGF-β treatment for 48 hr decreased the number of MPSC1 but not SBOT3.1 cells (Figure 1C). These TGF-β effects on apoptosis were in agreement with the invasion assay results.

### TGF-β induces phosphorylation of Smad3, but not Smad2, in SBOT3.1 and MPSC1 cells

**Figure 3:**
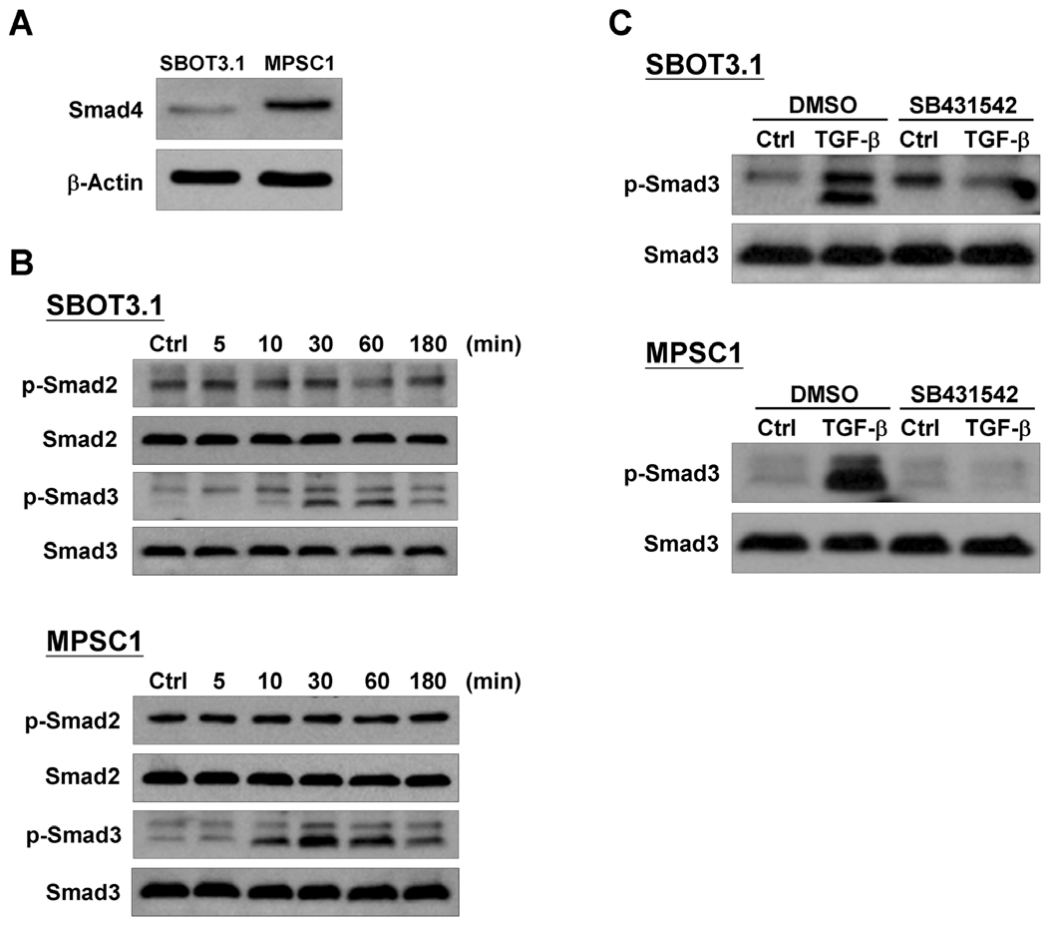
TGF-β induces the phosphorylation of Smad3 but not Smad2. A, The endogenous Smad4 protein levels were analyzed in SBOT3.1 and MPSC1 cells using a western blot. B, SBOT3.1 and MPSC1 cells were treated with 10 ng/ml TGF-β for the indicated durations. The Smad2 and Smad3 phosphorylation levels were determined using western blot analysis with antibodies specific for the phosphorylated, activated forms of Smad2 (p-Smad2) and Smad3 (p-Smad3). The membranes were stripped and reprobed with antibodies directed against Smad2 and Smad3. C, SBOT3.1 and MPSC1 cells were treated with SB431542 (10 µM) in the presence or absence of 10 ng/ml TGF-β for 30 min. The Smad2 and Smad3 phosphorylation levels were analyzed by western blot.

**Figure 4:**
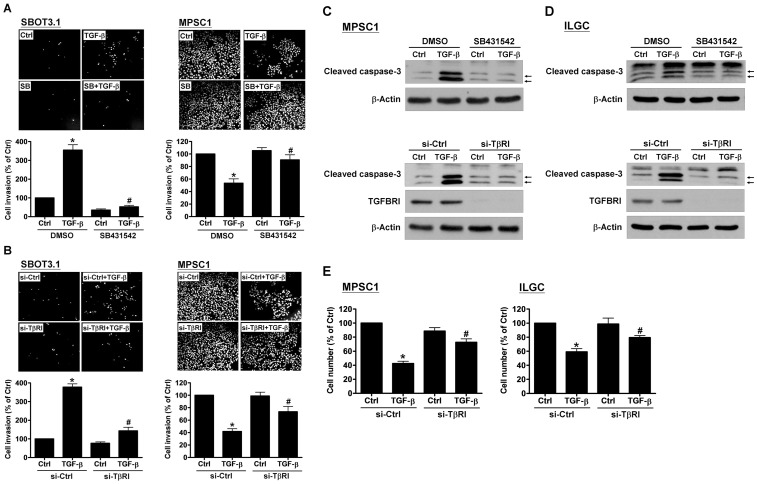
The specific TβRI inhibitor, SB431542, and TβRI siRNA abolished TGF-β-induced cell invasion and apoptosis. A, SBOT3.1 and MPSC1 cells were treated with 10 ng/ml TGF-β in combination with SB431542 (10 µM). B, SBOT3.1 and MPSC1 cells were transfected with 50 nM control siRNA (si-Ctrl) and TβRI siRNA (si-TβRI), and after 48 hr, the cells were treated with 10 ng/ml TGF-β. The cells were seeded onto Matrigel-coated transwell inserts. After 48 hr incubation, the non-invading cells were wiped from the upper side of the filter, and the nuclei of the invading cells were stained with Hoechst 33258. The results are expressed as the mean ± SEM of at least three independent experiments. C, MPSC1 cells were treated with 10 ng/ml TGF-β in combination with SB431542 (10 µM) for 24 hr or transfected with 50 nM control siRNA (si-Ctrl) or TβRI siRNA (si-TβRI) for 48 hr and then treated with 10 ng/ml TGF-β for 24 hr, and the levels of cleaved caspase-3 were examined western blot. Arrows indicate cleaved caspase-3. D, ILGC cells were treated with 10 ng/ml TGF-β in combination with SB431542 (10 µM) for 24 hr or transfected with 50 nM control siRNA (si-Ctrl) or TβRI siRNA (si-TβRI) for 48 hr and then treated with 10 ng/ml TGF-β for 24 hr, and the levels of cleaved caspase-3 were examined western blot. Arrows indicate cleaved caspase-3. E, MPSC1 and ILGC cells were transfected with 50 nM control siRNA (si-Ctrl) or TβRI siRNA (si-TβRI) for 48 hr and then treated with 10 ng/ml TGF-β for 48 hr, and the changes of cell number were examined using trypan blue exclusion assay. *p<0.05 compared with Ctrl in the DMSO group or Ctrl in the si-Ctrl group. ^#^p<0.05 compared with TGF-β in the DMSO group or TGF-β in the si-Ctrl group.

The Smad signaling pathway is important for regulating numerous TGF-β-mediated cellular functions. Western blot analysis demonstrated that the co-Smad Smad4 was expressed in both cell lines, although the level was higher in MPSC1 cells (Figure 3A). TGF-β treatment induced Smad3 phosphorylation in a time-dependent manner in SBOT3.1 and MPSC1 cells. However, in both cell lines, TGF-β did not alter the Smad2 phosphorylation level (Figure 3B). SB431542 is a potent and specific TβRI inhibitor [30]. Treatment with SB431542 significantly abolished the TGF-β-induced phosphorylation of Smad3 (Figure 3C) and the TGF-β-induced change in the cell invasion capability of SBOT3.1 and MPSC1 cells (Figure 4A). Moreover, the TGF-β effects on cell invasion were abolished by siRNA-mediated depletion of the TβRI receptor (Figure 4B). TGF-β-induced caspase-3 cleavage was eliminated by co-treatment with SB431542 and TβRI depletion using siRNA (Figure 4C). To further confirm the apoptotic effect of TGF-β in LGC cells, SV40 LT/ST immortalized LGC (ILGC) cells were used. As shown in Figure 4D, TGF-β treatment increased cleaved caspase-3 expression, which was abolished by SB431542 co-treatment and TβRI depletion by siRNA. In addition, TGF-β-decreased the MPSC1 and ILGC cell numbers were abolished by TβRI depletion by siRNA (Figure 4E).

### TGF-β induces EMT by up-regulating Snail, Slug, Twist and ZEB1 in SBOT3.1 cells

**Figure 5:**
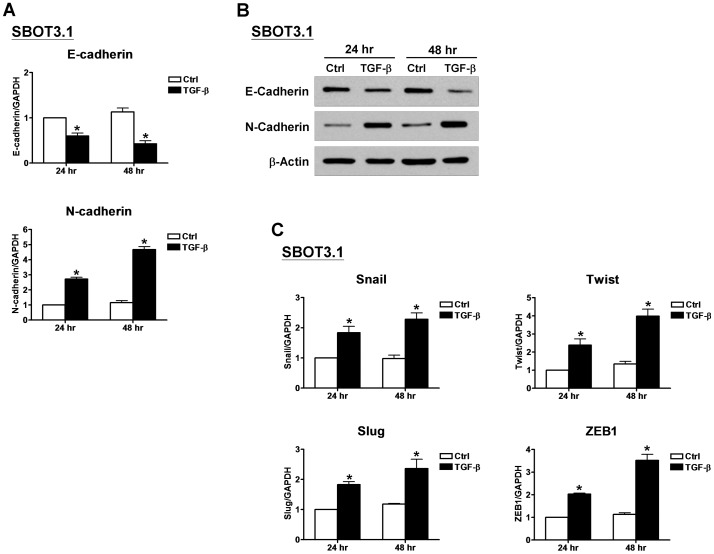
TGF-β induces a switch from E- to N-cadherin in SBOT3.1 cells. A, SBOT3.1 cells were treated with 10 ng/ml TGF-β for 24 and 48 hr. The E-cadherin and N-cadherin mRNA levels were analyzed by RT-qPCR. B, SBOT3.1 cells were treated with 10 ng/ml TGF-β for 24 and 48 hr, and the E-cadherin and N-cadherin protein levels were analyzed by western blot. C, SBOT3.1 cells were treated with 10 ng/ml TGF-β for 24 and 48 hr, and the Snail, Slug, Twist and ZEB1 mRNA levels were analyzed by RT-qPCR. The RT-qPCR results are expressed as the mean ± SEM of at least three independent experiments. *p<0.05 compared with time-matched control samples (Ctrl).

**Figure 6:**
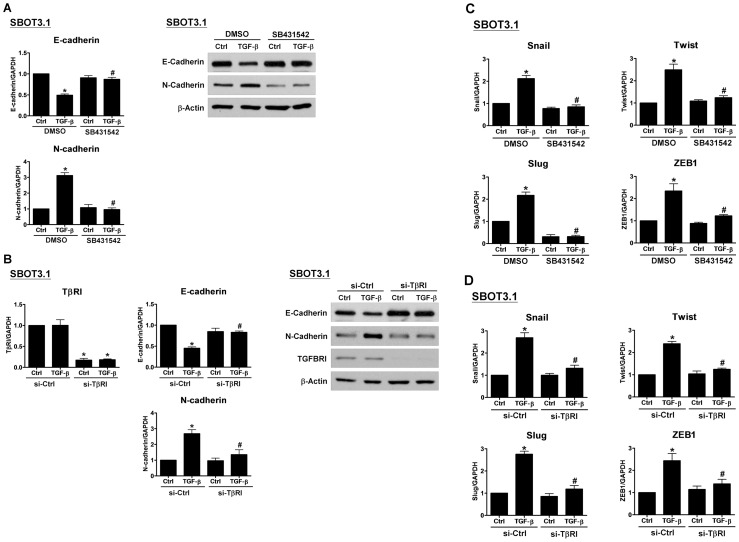
The specific TβRI inhibitor, SB431542, and TβRI siRNA abolished the TGF-β-induced differential change in E- and N-cadherin expression and the TGF-β-induced effects on Snail, Slug, Twist and ZEB1 expression. A, SBOT3.1 cells were treated with SB431542 (10 µM) in the presence or absence of 10 ng/ml TGF-β. E-cadherin and N-cadherin mRNA and protein levels were analyzed by RT-qPCR and western blot, respectively. B, SBOT3.1 cells were transfected with 50 nM control siRNA (si-Ctrl) or TβRI siRNA (si-TβRI), and after 48 hr, the cells were treated with 10 ng/ml TGF-β. The TβRI, E-cadherin and N-cadherin mRNA and protein levels were analyzed by RT-qPCR and western blot, respectively. C, SBOT3.1 cells were treated with SB431542 (10 µM) in the presence or absence of 10 ng/ml TGF-β. The Snail, Slug, Twist and ZEB1 mRNA levels were analyzed by RT-qPCR. D, SBOT3.1 cells were transfected with 50 nM control siRNA (si-Ctrl) or TβRI siRNA (si-TβRI), and after 48 hr, the cells were treated with 10 ng/ml TGF-β. The Snail, Slug, Twist and ZEB1 mRNA levels were analyzed by RT-qPCR. The RT-qPCR results are expressed as the mean ± SEM of at least three independent experiments. *p<0.05 compared with the Ctrl in the DMSO group or the Ctrl in the si-Ctrl group. ^#^p<0.05 compared with TGF-β in the DMSO group or TGF-β in the si-Ctrl group.

We next sought to better understand the mechanisms that mediate the TGF-β-induced EMT in SBOT3.1 cells. A switch from E- to N-cadherin expression has been suggested to be a key feature during EMT. RT-qPCR analysis demonstrated that TGF-β treatment down-regulated E-cadherin mRNA levels in SBOT3.1 cells while the N-cadherin mRNA levels increased (Figure 5A). These effects were confirmed at the protein level by western blot analysis following TGF-β treatment for 24 and 48 hr; SBOT3.1 cells demonstrated E-cadherin down-regulation and N-cadherin up-regulation at the total protein levels (Figure 5B). To investigate whether TGF-β down-regulates E-cadherin expression by modulating E-cadherin transcriptional regulation, we used RT-qPCR to examine the mRNA levels of the E-cadherin transcriptional repressors Snail, Slug, Twist and ZEB1. TGF-β treatment significantly increased the Snail, Slug, Twist and ZEB1 mRNA levels in SBOT3.1 cells (Figure 5C). SB431542 treatment and TβRI depletion by siRNA abolished the TGF-β effects on E- and N-cadherin mRNA and protein levels (Figures 6A and B). In addition, the TGF-β-induced changes to the Snail, Slug, Twist and ZEB1 mRNA levels were abolished by co-treatment with SB431542 and TβRI depletion by siRNA (Figures 6C and D).

## Discussion

SBOT and LGC have been recognized as entities that are distinct from HGC [31]. In this study, we used a cell culture system to demonstrate for the first time that TGF-β receptors are expressed in SBOT3.1 and MPSC1 cells. Interestingly, we also found that TGF-β exhibited a dual function whereby it induced SBOT3.1 cell invasion by activating an EMT, and it promoted apoptosis in MPSC1 cells.

Increasing evidence indicate that TGF-β functions as a tumor suppressor in early stage tumors while paradoxically acting as a tumor promoter in advanced cancers [6], [32]. The molecular nature of this switch is complicated, perhaps context dependent and remains largely unknown. In contrast to HGC, which presents as a clinically aggressive neoplasm that grows, rapidly spreads and is associated with poor outcome, LGC maintains its low-grade appearance and low proliferative index [18], [33] which may explain the TGF-β apoptotic effects on LGC cells. However, the role of this dual TGF-β function in the progression of SBOT to LGC requires further investigation. It has been recognized that the expression level of other endogenous factors present in tumor cells may affect the tumor cell autonomous switch of the TGF-β response from tumor suppressor to promoter [6]. LGC develop in a stepwise manner from OSE and SBOT [18]. p53 mutations are rarely detected in SBOT and LGC [18]. We have previously shown that p53 is wild-type in SBOT3.1 cells [24]. Microarray analysis of tumor specimens demonstrates that the p53 level is increased in SBOT compared to OSE, but the p53 level is decreased when SBOT progresses to LGC [34]. Our western blot results confirmed that the p53 level is higher in SBOT3.1 than MPSC1 (Figure S1). We have shown that TGF-β induces cell growth arrest in normal human OSE and SV40 large T immortalized human OSE (IOSE) [15]. Together with the current study, our results indicate that TGF-β functions as a tumor suppressor in OSE, IOSE, MPSC1 and ILGC, which all have low levels of wild-type p53 or inactive p53. In contrast, TGF-β acts as a tumor promoter in SBOT cells, which harbor high levels of wild-type p53. These results conflict with previous reports demonstrating that wild-type p53 is required for TGF-β-mediated growth arrest in normal mouse embryonic fibroblasts and hematopoietic progenitor cells [35]. In contrast, mutant-p53, but not wild-type p53, is required and can enhance TGF-β-induced breast cancer invasion and metastasis [35], [36]. In the SBOT/LGC system, the TGF-β switch from a tumor promoter to a tumor suppressor may be cell-type specific, or it may be affected by the endogenous level of wild-type p53. Further investigation will be needed to address this question.

A recent study demonstrated that Smad4 loss in colon cancer cells switches TGF-β from a tumor suppressor to a tumor promoter [37]. TGF-β induced proliferation, migration, invasion, tumorigenicity and metastasis in Smad4-null colon cancer cells, and these TGF-β-induced oncogenic effects were reversed when Smad4 expression was restored [37]. However, it is unknown whether Smad-dependent pathways are required for the observed oncogenic TGF-β effects in Smad4-null cells. Here we demonstrate that the endogenous Smad4 levels were lower in SBOT3.1 compared with MPSC1 cells. Whether the difference in Smad4 expression level affects the TGF-β functions in SBOT3.1 and MPSC1 cells will be an interesting topic for further investigation. Smad4 is essential for many but not all TGF-β-regulated transcriptional responses. In non-canonical signaling pathways, TGF-β activates MAPK, PI3K/Akt and small GTPases that are Smad-independent [38]. Our recent study demonstrated that the ERK1/2 and PI3K/Akt pathways are involved in EGF-induced EMT in SBOT cells [25]. Here we demonstrated that TGF-β treatment induced ERK1/2 and Akt phosphorylation in SBOT3.1 and MPSC1 cells (Figure S2). Because SB431542 is a TβRI inhibitor, it is not surprising that this compound completely blocked the effects of TGF-β on EMT and invasion in SBOT3.1 cells and apoptosis in MPSC1 cells. However, our results do not exclude the involvement of the non-canonical signaling pathways in TGF-β-induced EMT in SBOT3.1. Thus, further investigation will be needed to address this issue.

We now demonstrate that TGF-β treatment activates Smad3 but not Smad2 in SBOT3.1 and MPSC1 cells. Keratinocytes isolated from Smad2 knockout mice exhibit pathological alterations associated with EMT [39]. Furthermore, the absence of Smad2 in these cells leads to greater effects of TGF-β on Snail expression because of the increased availability of the Smad3/Smad4 complexes bound to the Snail promoter [39]. Smad2-deficient mouse hepatocytes acquire mesenchymal and promigratory features. In contrast, Smad3-deficient hepatocytes maintain their epithelial characteristics and do not exhibit TGF-β-induced apoptosis. These results suggest that Smad2 may have an antagonistic role in the induction of EMT, whereas Smad3 is required for TGF-β-induced EMT and apoptosis [40].

The transcription factors Snail, Slug, Twist and ZEB1 have been well characterized for their important roles in the regulation of the EMT through decreased E-cadherin expression [41]. In addition to their function in down-regulating E-cadherin, Twist and ZEB1 have been shown to up-regulate N-cadherin [42], [43]. Snail, Slug and Twist are involved in the TGF-β-induced EMT, which is mainly mediated by Smad3 [39], [44], [45]. TGF-β-induced Smad3 binds to the Snail and Slug promoters and activates their transcription [39], [45]. TGF-β can not induce Snail expression in renal tubular epithelial cells that are Smad3 deficient [46]. TGF-β also induces ZEB1 expression, although Smad3 involvement has not been reported [47]. MAPK signaling mediates ZEB1 expression, suggesting that a non-canonical signaling pathway may be involved in TGF-β-induced ZEB1 expression. In this study, TGF-β alone activated Smad3 and induced Snail, Slug, Twist and ZEB1 expression in SBOT3.1 cells, and these effects were abolished by the addition of the TβRI inhibitor SB431542. We have no direct evidence demonstrating that Smad3 is required for the TGF-β-induced expression of these transcription factors. Thus, additional studies will be required to examine the involvement of non-canonical signaling pathways in TGF-β-induced Snail, Slug, Twist and ZEB1 expression in SBOT cells.

In summary, we report for the first time the effects of TGF-β receptor expression in cultured SBOT cells and the LGC-derived cell line MPSC1. TGF-β treatment induces SBOT3.1 cell invasion by activating EMT. In contrast, TGF-β induces apoptosis in MPSC1 cells. Elucidating the functions of TGF-β in SBOT and LGC cells will increase our understanding of these particular types of human ovarian cancer.

## Supporting Information

Figure S1 **The endogenous p53 protein levels were analyzed in SBOT3.1 and MPSC1 cells using a western blot.**
(TIF)

Figure S2 **TGF-β induces the phosphorylation of ERK1/2 and Akt in SBOT3.1 and MPSC1.** Cells were treated with 10 ng/ml TGF-β for the indicated durations. The ERK1/2 and Akt phosphorylation levels were determined using western blot analysis with antibodies specific for the phosphorylated, activated forms of ERK1/2 (p-ERK1/2) and Akt (p-Akt). The membranes were stripped and reprobed with antibodies directed against ERK1/2 and Akt. Cells treated with EGF for 30 min were used as positive control.
(TIF)
